# Integrating Pharmacoproteomics into Early-Phase Clinical Development: State-of-the-Art, Challenges, and Recommendations

**DOI:** 10.3390/ijms18020448

**Published:** 2017-02-19

**Authors:** Savita Nandal, Tal Burt

**Affiliations:** 1 Novartis Pharmaceuticals Corporation, One Health Plaza, East Hanover, NJ 07936, USA; 2 Burt Consultancy, 2616 Erwin Rd, Durham, NC 27705, USA

**Keywords:** proteome, proteomics, drug development, early phase development, clinical development, biomarker, Omics, clinical research, phase 0, phase 1, phase 2, proof-of-concept, proof-of-mechanism, proof-of-principle, first-in-human (FIH) studies, pharmacogenomics, pharmacometabolomics, pharmacokinetics (PK), pharmacodynamics (PD), drug toxicity

## Abstract

Pharmacoproteomics is the study of disease-modifying and toxicity parameters associated with therapeutic drug administration, using analysis of quantitative and temporal changes to specific, predetermined, and select proteins, or to the proteome as a whole. Pharmacoproteomics is a rapidly evolving field, with progress in analytic technologies enabling processing of complex interactions of large number of unique proteins and effective use in clinical trials. Nevertheless, our analysis of clinicaltrials.gov and PubMed shows that the application of proteomics in early-phase clinical development is minimal and limited to few therapeutic areas, with oncology predominating. We review the history, technologies, current usage, challenges, and potential for future use, and conclude with recommendations for integration of pharmacoproteomic in early-phase drug development.

## 1. Introduction and Background

Drug development is a complex and challenging process and is becoming more so, with increasing costs and reduced productivity [1,2,3]. Clinical development in particular is becoming more expensive and prone to failure [4,5,6,7]. “Omics” approaches provide an opportunity to accelerate drug development in general and clinical development in particular [8,9,10]. By providing biomarkers associated with drug response and illness progression, “Omics” approaches provide efficient and convenient surrogate endpoints as outcomes of clinical trials [11,12,13]. This is of particular value in early-phase clinical trials which are typically short, small-sized, and consequently underpowered [14,15,16]. We previously reviewed the role of pharmacogenomic and pharmacometabolomic approaches in early-phase clinical development and here we review the role of pharmacoproteomics [14,15].

Genomics, transcriptopmics, proteomics, and metabolomics constitute the study of indigenous biological entities on a continuum of code, production, action, and by-product generation, respectively. These molecular entities are part of a continuous, ontological, and iterative process that represents the organism’s interactions and adaptations to internal and external survival events including growth, nutrition, illness, and healing. Genomics is the study of the function and structure of the complete genome. The proteome constitutes the aggregate of proteins, including receptors and enzymes, and is the action medium of the “Omics” space. Metabolomics is the study of the metabolome, which is the end-product of the genome translated into proteins + proteomic activity + results of proteomic and genomic interactions with multiple external and internal factors. The proteome is the snapshot of proteins in action, at the time of measurement. The systematic study and profiling of all the organism’s proteins including their structure, function, and interactions, is “proteomics”. Proteomics play an essential role in the comprehensive understanding of the organism’s functions, interactions, and adaptations that “systems biology”, or “Omics” sciences, aim to provide.

Multiple factors influence proteins expression and their effects in the organism. Improving methods and technologies have enabled the discipline of proteomics to move from single protein assessments to multiple and whole-body comprehensive protein assessments or proteomics. In general, proteomic information is greater and more complex when compared with genomics as post-translational events can lead to production of millions of unique proteins from the roughly 20,000 human genes that code for them. Protein function measurements are particularly quite challenging. The past 2 decades have witnessed considerable progress in proteomic technologies improving traditional separation and mass spectrometry, allowing complex mixture analysis, and introducing microarrays, chip based approaches and other functional and affinity-based approaches (See Box 1). A draft map of the human proteome is currently available utilizing high-resolution mass spectrometry [17]. With improving technologies and techniques, it is becoming increasingly feasible to profile the individual human proteome and include such analyses in clinical trials. There has been a nonsystematic utilization of proteomic approaches in drug development with studies predominantly conducted in oncology.

The interaction of every drug with the human proteome is complex and diverse. Drugs may affect protein-protein, protein-nucleic acid, other protein interactions or post translational modifications, resulting in desirable and undesirable actions in the human body. The interaction may affect multiple entities contemporaneously and change with time in response to internal and external conditions. The integration of proteomic (and other “Omics”) approaches early in the drug development process provides an opportunity to develop, validate, and then utilize the biomarkers to study specific drug effects in well-characterized patient populations, as well as explore yet unknown efficacy and toxicity targets and outcomes. Proteomic approaches may assist early identification of proteome diversity in response to therapeutic drug interventions, lead to better development decisions, and potentially facilitate development of drugs with better efficacy, safety, and tolerability profiles, and accelerate developmental timelines.

This review describes the role and utilization of pharmacoproteomics in early-phase clinical development (the first-in-human (FIH) and other early, small, clinical development trials) and its potential to facilitate translational effectiveness in drug development. We include assessment of utilization of pharmacoproteomics in the early-phase clinical development using an analysis of clinicaltrials.gov and PubMed databases, review relevant examples, discuss related challenges and opportunities, and conclude with recommendations for future development of the field.

## 2. History

Berzelius used the word protein in 1838. Single protein assessments have played an important role in the diagnosis and management of various diseases. Progress in the field usually took place in tandem with development of analytic technologies and, more recently, bioinformatics. Protein analysis was introduced in 1975, with the use of two-dimensional gel electrophoresis (2DE), and clinical studies started using such analyses in 1980’s. In 1995 Wasinger et al. [18] coined the term proteome to describe the protein complement of the genome. First use of isotope coded affinity tags (ICAT) in a proteomic study was reported in 1999 expanding the range of proteins that could be studied thus allowing a less biased mode of proteomic study. More recent developments in mass spectrometry and bioinformatics have enabled simultaneous analyses of large numbers of proteins in complex mixtures, making the use of proteomics as biomarkers in clinical trials increasingly feasible and effective.

The term pharmacoproteomics was first used by Kennedy et al., and Meister et al. in 2002 [19,20], however a standard definition does not exist yet. We propose the following definition: “Pharmacoproteomics is the study of disease-modifying and toxicity parameters associated with therapeutic drug administration, using analysis of quantitative and temporal changes to specific, predetermined, and select proteins, or to the proteome as a whole”. Table 1 includes historical milestones relevant to pharmacoproteomics and Box 1 includes more detailed descriptions of the key analytic techniques used in proteomic studies. Pharmacoproteomics has a role to play in all the phases of translational research as the drug moves from discovery, to humans, to patients, to clinical practice and to the communities. The term “chemoproteomics” is sometimes used to describe the pharmacological aspects of the interactions of proteins with other molecules, including drugs, and the relevance of such interactions to therapeutic efficacy and toxicity [21,22].

Notwithstanding the progress in associated technologies, increasing feasibility, and inclusion in clinical development very few proteomic biomarkers have been able to establish clinical utility and receive regulatory approval in clinical practice [11,23]. Success with proteomic applications has been limited and slow predominantly due to the exploratory and retrospective nature of many proteomic studies, the complexity and large number of proteomic interactions, and the challenges of processing large amounts of data needed to identify and validate relevant biomarkers [13]. These challenges are gradually being addressed with more sensitive, comprehensive, minimally-biased, and fast analytic approaches in tandem with sophisticated bioinformatics approaches required to process, analyze and store the large amounts of data. These advances will likely gradually increase the utility and inclusion of proteomics and other “Omics” biomarkers in translational and clinical studies.

## 3. Pharmacoproteomics in Early Phase Development

Early-phase clinical development comprises the typically small (*n* < 80) and short (days to weeks) clinical trials in healthy volunteers or patients, which constitute the first studies in humans of a new drug, or the study of an existing drug in a new population or new indication. Such studies provide initial proof-of-mechanism (POM) and proof-of-concept (POP) information on drug pharmacokinetics (PK), pharmacodynamics (PD), and safety. Information obtained will be confirmed in later-stage definitive registration studies with typically larger sample size (*n* > 300) and duration (months to years).

Recent advancements in proteomic technologies, bioinformatics and data management have enabled the transition of proteomic approaches from exploratory research tools into routine and efficient clinical development tools. Use in early-phase clinical studies can take two general forms: use of already validated proteomic markers to study the drug being developed or the continued development and validation of proteomic biomarkers so they can be used in later stage development and future development programs. Early-phase studies are an optimal time to use proteomic (and other “Omics”) biomarkers to study drug effects because relatively little is known at the time about drug effects in humans and restricted size, duration, and populations of these studies limit study power and the ability of traditional clinical outcomes to detect true effects, both positive and negative. Proteomic approaches can help reduce uncertainty about pathways, mechanisms, pharmacokinetics, and preliminary signals of response diversity and help optimize the larger, longer, and more expensive later-phase studies.

It is critical to plan and initiate the integration of proteomic biomarkers into development programs as early as possible and preferably no later than the time of candidate selection (See under “recommendation” below for details). Strategic and methodological details should be discussed with regulatory authorities, and analytic, bioinformatics, and sample-management infrastructure established. Application of pharmacoproteomics at early phase trials in the clinical development provides an array of benefits with specific examples from our PubMed search outlined in Table 2 and below. But perhaps most importantly, well-validated, sufficiently sensitive and specific biomarkers capable of detecting the “true negative” drugs in early trials, could lead to early termination of expensive and futile development programs, and could channel resources sooner to alternative back-up candidates thereby optimizing their patent-life [53].

Specific examples of studies that successfully applied pharmacoproteomic principles in early-phase development were identified through structured PubMed search and are listed in Table 2. Such uses provide several potential advantages:

Target validation: this is possibly the most important contribution of pharmacoproteomics to early-phase clinical development due to the potential for early termination of the development of ineffective or toxic compounds. Understanding targets’ roles in normal physiology and disease pathogenesis and information on the biological mechanisms leading to response and toxicity could be helpful in target validation. In a recent study conducted by Cheraghchi-Bashi et al., proteomic signatures identified pre-clinically were validated and provided insight into biological mechanisms in a phase 1 study [55].

Biomarker development and validation: early-phase studies are the first opportunity to test in humans, continue development, and validate proteomic biomarker candidates that were identified in pre-clinical work. Such validation is perquisite of the utilization of such biomarkers in later-phase studies and also provides the opportunity to generate hypothesis for further biomarker development studies [75]. Samples for biomarker validation should be part of carefully planned prospective studies preferably after consultation with regulatory authorities [8].

Pharmacokinetics (PK) and pharmacodynamics (PD): drug exposure and disposition may affect drug interaction with the proteome and vice versa, the proteome (e.g., through protein binding and metabolizing enzymes) may impact drug PK and PD properties. Understanding this complex and fluid interaction may be crucial to elucidating drug efficacy and toxicity effects and is important to establish as early as possible in clinical development. In addition, understanding the proteomic profile of PK and PD outliers may help better characterize target populations for later-phase studies. Help with therapeutic window assessments, interaction with blood brain barrier, drug-drug interactions (DDI), dose-response and concentration-response relationships relevant to the drug disposition or disease under study, are other examples of the potential utility of proteomics in the study of drug properties and effects [75].

Insight into the impact of variability on pharmacotherapy outcomes: pre- and post-treatment proteomic data could distinguish responders from non-responders and possibly identify reasons for outcomes heterogeneity. Proteomic diversity could lead to variability in drug responses and present the opportunity to identify efficacy and toxicity phenotypes unique to specific populations [62,66,72].

Better Study design for larger clinical trials: Proteomic signatures could assist with streamlining clinical development plans by optimizing inclusion and exclusion criteria, and enabling patient stratifications in larger later-phase studies [62,66,72]. Stratification causes study populations to be more homogeneous. The reduced variability translates into smaller sample size requirements and better chance of demonstrating efficacy and tolerability, however with the limitation of use in a more restricted patient population.

Reduced sample sizes: using proteomic biomarkers as surrogate endpoints could increase the effect size of drug effects in early-phase trials, reduce sample size requirements, and increase the efficiency of clinical development programs [76]. This could be of particular value in drug development for rare diseases where recruitment presents a challenge and sample sizes are typically low.

Drug response and resistance: proteomic approaches could help elucidate mechanistic principles of drug response and drug resistance. This could be useful for identifying potential drug combinations and back-translation of the findings to enable identification of new targets and development of follow-up compounds [58,72,77]. Proteomic signatures of efficacy may help reduce variabilities associated with placebo response.

Early information on assay development and feasibility: early-phase studies can provide crucial information on feasibility of assays before implementation in larger later-phase studies [54]. Proteomic biomarkers are generally easily accessible through body fluids or tissue biopsies and can often be collected noninvasively with multiple samples collected over any required time course.

Compliance: proteomic makers of disease states and drug effects could be used to assess compliance in clinical trials and could be especially useful for monitoring drugs with narrow therapeutic window.

Accelerated clinical development and registration timelines: better study designs and smaller study sample sizes could help identify subpopulations experiencing benefits or risks, and lead to accelerated clinical development timelines. Early information on responders and specific value of the drug in specific population could enable faster approvals for specific indications supported by biomarker data [78].

Ethics: proteomic approaches, in line with the promise of personalized medicine, could enable more ethical study designs by identifying subject populations with optimal benefit/risk ratio. This could be done by excluding those unlikely to respond to the drug or those more likely to experience adverse effects. Once studies are initiated, proteomic biomarkers could contribute to the ethical conduct of studies by limiting duration of exposure to ineffective drugs, and identifying early signs of toxic potential. Overall, increasing the efficiency of drug development could lead to quicker delivery of new therapeutics.

## 4. Clinicaltrials.gov and PubMed Analyses

### 4.1. Purpose

Clinicaltrial.gov and PubMed searches were conducted to determine the utilization of proteomics in early-phase clinical trials (phase 0, 1, or 2 studies).

### 4.2. Methodology

Clinicaltrials.gov database was accessed on 27 November 2016 using the keyword “proteomic” and filters for “interventional studies”, and “phases 0, 1, or 2”. PubMed search was conducted on 18 November 2016. The keyword used was “proteomic analysis” and the search was refined with selection of phase 1 and 2 clinical studies (there was no option for choosing Phase 0 studies). Each result entry was independently reviewed by the two authors (Tal Burt and Savita Nandal.) to confirm that it met selection criteria. Any discrepancies between the authors’ assessments were reconciled in a consensus discussion.

### 4.3. Results

**Figure 1:**
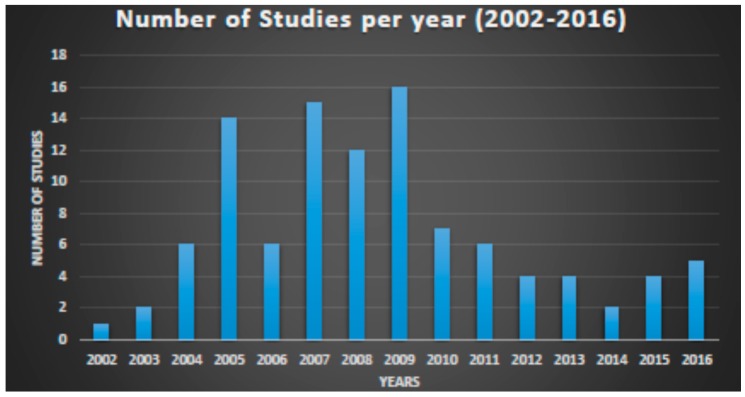
Number of early phase clinical trials per year, using proteomic approaches. Data from clinicaltrials.gov 2002–2016.

**Figure 2:**
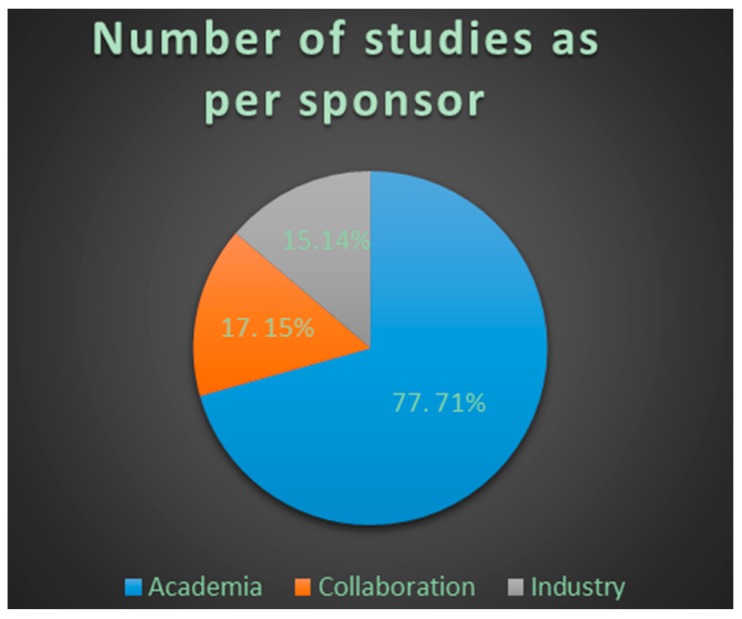
Number and percentage of trials per sponsor using proteomic approaches in clinical studies. Data from clinicaltrials.gov 2002–2016. Collaboration represents clinical studies conducted by academic institutes in collaboration with industry.

**Figure 3:**
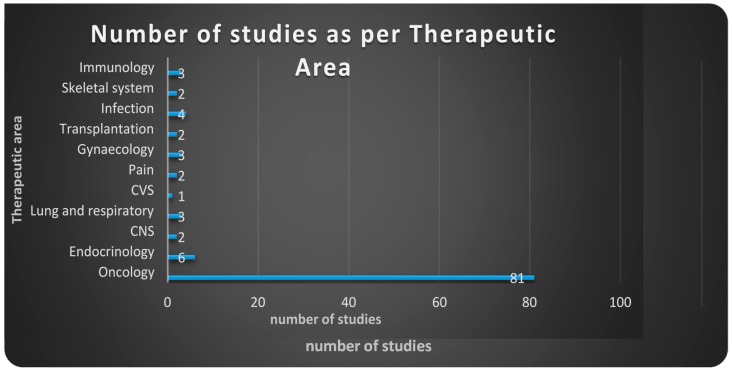
Number of studies as per therapeutic areas utilizing the proteomic approaches. Data from clinicaltrials.gov 2002–2016.

Clinicaltrials.gov database Analyses (Table A, Supplementary Materials). From 123 search results a total of 109 were identified as early-phase studies with proteomic biomarkers as one of the outcomes. This represents 0.14% of the 76,188 early-phase interventional studies in the database. First use of proteomics in early-phase studies was noted in 2002. Over the past 14 years there has been initially a gradual increase in pharmacoproteomic early-phase studies followed by a decrease after 2009 (Figure 1). The majority of studies (77; 71%) were conducted by academic institutions (17; 15%) were conducted by industry, and (15; 14%) were industry-academia collaborations (Figure 2). Oncology was the most common therapeutic area in more than 80% of studies. Other therapeutic areas with less than seven studies each were endocrinology, gynecology, respiratory, immunology, transplantation, cardiovascular, central nervous system, infectious diseases, skeletal system and pain (Figure 3).

Pubmed analysis (Table 2). From 45 search results a total of 21 manuscripts were identified as pharmacoproteomic early-phase trials. This represents 0.056% of the 37,919 early-phase publications in PubMed. First mention of an early-phase pharmacoproteomic study was in 2006. Although the total number of reported studies in PubMed is too small to detect any meaningful trend in use there were 7 publications in the period 2006–2010 and double (14) in the 2010–2016 period. The therapeutic area in 20 reports was oncology with one study in infectious disease.

### 4.4. Conclusions

Very few studies have applied proteomic approaches in early-phase clinical development with similarly low percentages reported in clinicaltrials.gov and PubMed (0.14% and 0.056%, respectively). Most of the studies are being conducted by academic institutions as sponsor (84.4%) with limited utilization by industry. After increase in utilization in the 2005–2009 period there appears to be some decline in use in recent years. The therapeutic area with most common utilization is oncology.

### 4.5. Limitations

Our search strategy was dependent on the inclusion of the term “proteomic” in clinicaltrials.gov and PubMed entries. It is possible that studies used proteomic biomarkers but have not identified them as such in keywords or MeSH terms. In addition, studies conducted prior to phase 2 (i.e., phase 0 and phase 1 studies) are not required to be registered in the public domain and may have not been included in the clinicaltrials.gov database [79]. This may have exposed our analysis to reporting bias.

### 4.6. Challenges

The application of pharmacoproteomics has the potential to add numerous advantages to the drug development process including early-phase trials. However, it comes with methodological, operational, regulatory, financial, ethical and legal challenges (Table 3). Understanding the challenges will help plan and address them proactively, minimize, and mitigate any negative impact [80,81].

Expertise on the evolving proteomic technologies and understanding on the maturity of the technology with their limitation is challenging and complex.

The application of pharmacoproteomics increases protocol and study design complexities and requires meticulous planning. The complexity increases due to the need to have validated assays and associated sample collection, processing, storage, statistical modeling, and bioinformatics infrastructure [13]. Multiple hypotheses may have to be tested in one clinical trial [82]. Consent forms are more complicated and additional information on sampling/biopsies, biobanks, data utilization, and privacy concerns has to be included [83]. Operational burden on study sites and participating study subjects increases with additional sampling requirements and procedures related to sample collection, processing, storage, and shipping. Participant burden may translate to enrollment challenges. Regulatory burden increases, with requirements to comply additional rules and regulations for biomarker development. In global multicenter trials where export of samples to central laboratories is required, timelines may extend due to approval requirements for the export process. Collaborations with external partners may be required in absence of specific on-site or intra-organization expertise related to biomarker and assay development. Intellectual property is to be handled carefully and is to be done early during the development process and clarified with collaborators [82]. Overall budget for early-phase development may become substantially higher due to these various challenges.

### 4.7. Recommendations

**Figure 4:**
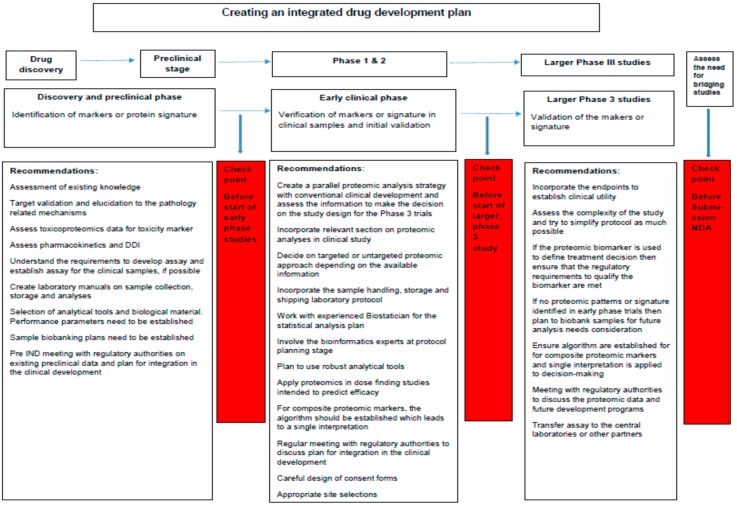
Recommendation on integrating pharmacoproteomic approaches in clinical development plan. The figure illustrates the checkpoints along the conventional clinical research continuum where pharmacoproteomic biomarkers development and application should be assessed.

Application of pharmacoproteomic approaches in clinical development plan can be both challenging and rewarding. Understanding the entire process and applying it in a meticulous and informed manner is essential. Incorporating proteomic analyses into prospective, randomized, controlled trials represent an opportunity to increase the value of the data obtained and efficiency of the development program in general (see Figure 4). Proteomic analyses can help create a more dynamic clinical development program as compared to the conventional drug development process. The information generated during the early-phase studies could change the target populations, endpoints, dose selection, risk mitigation plans and could possibly repurpose drug therapeutic targets.

Involvement of regulatory authorities in the planning stage and self-regulation at various checkpoints is recommended [13]. Utilization of validated analytical tools and bioinformatics is of outmost importance and use of qualified central laboratories for analytics, simulation studies for guidance into the study design, which involves high-dimensionality data is advised [84]. It is essential to establish detailed standard operating procedures (SOPs) for all the steps involved in proteomic analyses including sample collection, storage, transfer, quality standards and analysis. It is likewise important to follow reporting guidelines for various standards and have a clear strategy on validation process that should be carefully designed and executed. The choice of clinical sites for early-phase studies has to be done carefully to ensure adequate matching of site expertise and infrastructure with proteomic protocol elements. Meticulous attention to execution details at every step is needed, and it is recommended that regular monitoring of proteomic procedures take place and ad-hoc training provided if needed.

Involvement of various stakeholders including basic scientists, statisticians, bioinformatics, analytic experts, clinical researchers, regulatory authorities and patient advocates will ensure optimal planning, execution, and interpretation of proteomic data. It is important to have the appropriate study design. In phase 1 studies, application of pharmacoproteomics is done in open label dose finding studies where proteomic biomarkers could either be used as an exploratory or secondary endpoint depending on the existing pre-clinical data and degree of biomarker development and validation. Innovative trial designs have been utilized in the omics field and could be applied for proteomics too [85]. Phase 2 studies should be able to guide the study design and assist with informed decision making on conduct of phase 3 studies example whether biomarker enrichment or stratified design should be implemented, if biomarker strategy should not be involved, or if phase 3 is not required at all.

**Figure 5:**
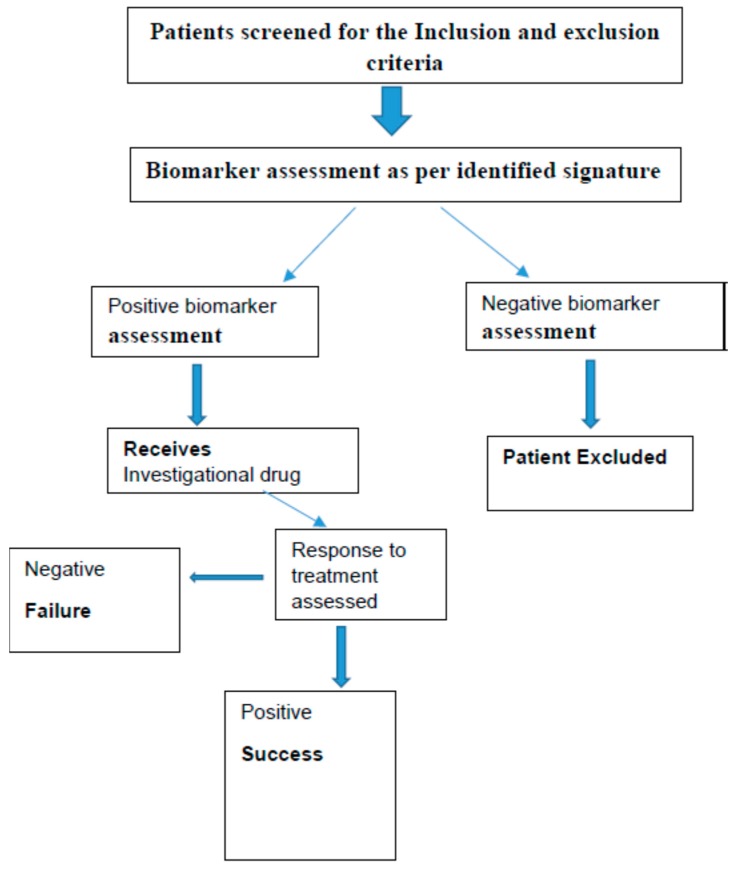
Single arm, open label—one stage biomarker enriched design approach.

**Figure 6:**
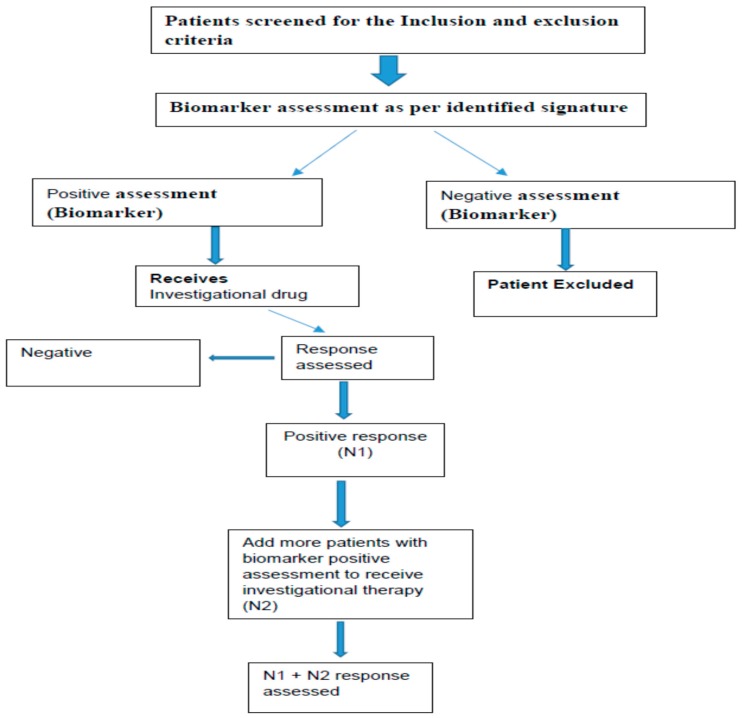
Open label with two-step biomarker enriched design approach.

The study designs, which are commonly used for the phase II are generally open label biomarker enriched designs [86,87,88]. Examples schematically shown in Figure 5 and Figure 6. There are many other designs like adaptive parallel two stage design, Phase 2 with randomization, Phase I/II adaptive design, Phase II/III adaptive designs and prospective-retrospective design [88,89].

All designs have their pros and cons and the choice to biomarker enrich design will depend on the available data, resources, complexity and on the objective of the study [90].

Using proteomic principles in clinical development studies gives a unique opportunity to identify and develop new biomarkers in prospective studies with well-defined populations. Such inclusion in clinical trials is a necessary step in completing the validation process, gives developers confidence regarding differing drug effects in sub-populations thus helping forward the promise of precision medicine. This could accelerate developmental decisions and regulatory processes, bring better therapeutic and outcome assessment solutions to patients and their caregivers, and provide individualized information to payers to increase the accuracy of reimbursement. Study designs should be well thought through and ensure that drug effects are not obscured by potential heterogeneity in study populations. Incorporating proteomic analyses into study design is an expensive and time-consuming endeavor and careful planning, qualification and validation of the markers, establishing clinical utility plans, sample processing infrastructure, and specialized expertise necessary to process and interpret the data, are all required. A proposed integrated plan is schematically shown in Figure 4.

## 5. Conclusions

The proteome reflects the dynamic interactions with self, pathology, and external environment. Proteins are modified to adapt to new stresses and requirements by a variety of post-translation modifications. Pharmacoproteomics is an important tool in the drug development process with potential to streamline clinical development plan by providing strategies to decrease heterogeneity, and prognostic and predictive enrichment designs. Early-phase development is an opportune time where the hypotheses could be generated and tested for later application in large prospective study. Nevertheless, the utilization in early phase development is miniscule with limited application in few therapeutic areas such as oncology. Understanding the challenges could assist drug developers in apply proteomic principles in a standardized and effective manner. We recommend integrating pharmacoproteomic applications into conventional drug development prior to initiation of first-in-human trials. Early understanding and strategic planning of proteomic biomarker development and validation including specialized versions of the following: biostatistics, analytics, bioinformatics, sample collection, processing, sample biobanks, and study designs. Working with all relevant stakeholders including basic scientists, regulatory agencies, patient advocacy groups, clinical researchers, technology and information processing experts, is imperative. Challenges associated with logistic, temporal, and resource investment in pharmacoproteomic approaches are likely to be compensated by reduction in uncertainty about drug effects and acceleration of developmental decisions.

### 5.1. Box 1: Analytic Approaches Used in Proteomic and Pharmacoproteomic Studies

Proteomic analytic technologies are tasked with 5 main objectives: separation (sometimes called resolution or fractionation), mass analysis (allows protein identification), abundance determination (quantification), protein structure, and function. The first two techniques, separation of proteins from complex biological samples, and determination of their individual masses, have been in use since the 1980’s, mainly in the form of 2-dimentional gel electrophoresis and mass spectrometry, respectively [91]. Since the early 2000’s additional technologies emerged to provide the crucial information about protein abundance, structure, and function. These approaches include: multidirectional protein identification technology (MudPIT) [92,93], isobaric tags for relative and absolute quantification (iTRAQ) [50], stable isotopic labeling by amino acids in cell culture (SILAC) [50,51,94], yeast two-hybrid systems [49], isotope coded affinity tags (ICAT) [48], protein chips [95], and activity-based probes (ABPs) [96].

Considerable progress in automation, statistical and bioinformatics approaches has been made to allow effective and timely analysis of large numbers of protein in complex mixtures over multiple time points.

Protein analysis presents several challenges. Protein concentrations are continuously fluctuating in response to a variety of internal and external factors, physiological and environmental in nature, and may not reliably reflect protein activity. In addition, protein-protein interactions as well as interactions with other molecules may affect protein concentrations and activity. The often very low concentrations may challenge the limits of sensitivity of analytical instruments, and notably, protein levels of expression and concentrations are not necessarily reflective of their function. Finally, the large number of proteins, much larger than genes and well into the millions due to the many possible post-transcription modifications, make the identification and quantification of proteins that are relevant to drug effects difficult.

#### 5.1.1. 2D Gel Electrophoresis (2DE) [91]

The technique uses two-dimensional electrophoresis method to separate (fractionate) proteins. In one dimension proteins are separated by their electric charge and in the second dimension by their molecular weight with those with higher charge and lower mass proceeding further in the gel medium. The technique has limited sensitivity and separation effectiveness. It also does not provide quantitative or functional information about the proteins.

#### 5.1.2. Mass Spectrometry [92]

Mass spectrometry (MS) was traditionally used together with 2DE to identify individual proteins in biological samples. MS measurements take place on ionized compounds in the gas phase. The ionized proteins or protein fractions are passed through a mass analyzer that deflects them based on their mass and charge to generate unique mass-to-charge ratio beams that are then counted by a detector. Protein transformation to the gas phase and ionization are performed using either matrix-assisted laser desorption/ionization (MALDI) [97], Surface-enhanced laser desorption/ionization (SELDI) [44], or Electrospray ionization (ESI) [98].

MALDI-MS uses laser pulses to sublimate and ionize proteins in a crystalline matrix before running them through the MS [44]. MALDI-TOF (time-of-flight) is a MS approach that distinguishes compounds based on the time it takes them to reach the detector. The ionized peptides are accelerated in strong electric field. Heavier compounds take longer to reach the detector.

SELDI-MS is a variation on MALDI that uses protein attachment to a surface as the sample-presenting medium prior to MS analysis.

ESI-MS vaporizes and ionizes proteins from a liquid source, best suited for use with liquid chromatography (LC) modes of protein separation and optimal for analysis of complex protein mixtures. ESI-MS was introduced in 1989, at about the same time as MALDI for the analysis of large biomolecules including proteins [43].

#### 5.1.3. ELISA and Immunohistochemistry

These approaches use antigen-antibody affinity as the basis for identification of proteins in liquid mediums or tissue sections, respectively [26,29,30]. These are targeted approaches that require pre-existing analytic tests to known proteins. Enzyme-linked immunosorbent assay (ELISA) introduces sample proteins (as antigens) from a liquid medium to a surface that causes them to become attached to it. An antibody attached to an enzyme is then introduced and the antigen-antibody reaction activates the enzyme and this leads to some measurable physical property (e.g., fluorescence). Immunohistochemistry uses similar principles but keeps the original structure of the tissue in histological samples allowing location of the proteins in tissue architecture.

#### 5.1.4. Multidirectional Protein Identification Technology (MudPIT) [92,93]

The technology takes complex mixtures of peptides from digested proteins and then separates them using microcapillary LC column with strong cation exchange (SCX) and reverse-phase (RP) material. The separated products are then passed directly into the MS machine for analysis. Sometimes described as a “shot-gun” approach it is a non-targeted high throughput method of separating and identifying large numbers of proteins. The approach is largely unbiased, meaning that it is not limited to narrow ranges of abundance, molecular weight, isoelectric point, and hydrophobicity as traditional 2-dimentional gel polyacrylamide electrophoresis (2D-PAGE) were [92]. Nevertheless, the approach does not provide information about protein abundance and function.

#### 5.1.5. Isobaric Tags for Relative and Absolute Quantification (iTRAQ) [52]

iTRAQ is a quantification method for proteins in complex mixtures that allows both relative and absolute measurements. Isobaric, meaning of equal mass, tags are applied to peptide mixtures and attach to their N-terminus and side-chain amines. The tag is fragmented during mass spectrometry to generate a reporter ion that allows for precise quantification. The labeling tags can be applied to peptides from both cell culture and tissue sources.

#### 5.1.6. Stable Isotopic Labeling by Amino Acids in Cell Culture (SILAC) [48,49,78]

This approach is used for analyzing proteins in cell cultures by incorporating stable isotopes into amino acids, often arginine and lysine, and then allowing different cell cultures to grow with different labelled amino acids. For example, one culture may use amino acids with ^12^C or ^14^N and the other ^13^C or ^15^N, respectively. The different isotopes behave chemically the same and have no impact on the function of the proteins they are part of. The difference in isotope physical properties, however, allows sensitive detection of protein abundance and is used to compare proteomes from cells that were grown under different conditions.

#### 5.1.7. Isotope Coded Affinity Tags (ICAT) [50,51]

Isotopic labeling allows for highly sensitive detection and quantification of proteins. ICAT was used to identify factors that alter drug sensitivity in the treatment of colorectal cancer [98].

#### 5.1.8. Functional Proteomic Analyses [12]

Since most proteins are involved in physiological activities an important component of protein characterization is their function. Protein microarrays, or protein chips, allow high throughput detection of proteins of interest, including interactions with other proteins, phospholipids, and small molecules [95,99,100]. The disadvantages of such approaches is that prior knowledge of the targeted proteins is required to develop the array and the production and validation of new arrays is complex. Yeast two-hybrid systems identify protein function through a “lock and key” matching. If a match occurs it start a chain reaction that leads to a macro signal, such as change of color, confirming the presence of the hypothesized function [49]. Other functional tests exist, such as phage and ribosomal displays that use protein-DNA and protein-mRNA interactions, respectively to identify specific proteins [101,102].

#### 5.1.9. Activity-Based Protein Profiling [96,103,104]

Activity-based proteomics uses small molecule probes called activity-based probes (ABPs) that covalently attach (i.e., tag) and modify the active sites of proteins (usually enzyme) [100]. This allows detection of and purification of the target enzyme. A main advantage of ABPs is that they account for post-translational modifications [12]. It also provides a method for study of the impact of drugs on proteins, especially on off-target adverse events [105,106].

#### 5.1.10. Reverse Phase Protein Arrays (RPPA) [107,108]

One of the platforms for protein analysis, used for quantitative assessment of proteins and phosphoprotein in the cell and tissue lysate. The steps involved are preparation of protein lysate, and then the dilutions using liquid handling systems, robotic arrayers used for spotting and signal detection conducted using the validated antibodies. Finally, RPPA software used for the analysis. This technology has many advantages as high throughput, sensitive, low amount of sample requirements and allows assessment in multiple samples. The first use of this method reported in 2001 and now frequently used in the research and clinical setting. The only limitation of this technology is the requirement for the specific antibodies for each assay.

## Figures and Tables

**Table 1:** Historical milestones relevant to pharmacoproteomics.

Milestone	Year	Description	References
The word “protein” first used	1838	Swedish Chemist Jöns Jakob Berzelius	[24]
Mass spectrometer	1913	J.J. Thomson constructs the first mass spectrometer	[25]
Immunohistochemistry	1941	First description of the methodology	[26]
LC, GC, and MS in biology	1960s	liquid (LC) and high-performance liquid chromatography (HPLC), gas chromatography (GC) and mass-spectrometry (MS) used to characterize physiologic and pathophysiologic states (quantitative)	[27,28]
Enzyme-linked immunosorbent assay (ELISA)	1971	First description of the methodology	[29]
			[30]
Introduction of two-Dimensional gel	1975	The first protein studies that can be called proteomics began in 1975 with the introduction of the two-dimensional gel and mapping of the proteins from the bacterium Escherichia coli, guinea pig and mouse	[31]
			[32]
			[33]
Combining LC with MS = LCMS	1980s	First interfaces for combining liquid chromatography with mass spectrometry (LC-MS) emerge	[34]
			[35]
			[36]
			[37]
Attempt to catalog human proteins	1980	Attempt to catalog human proteins	[38]
2-Dimentional electrophoresis (2DE)	1984/5	First studies to use 2DE for human protein separation	[39]
			[40]
Development of microsequencing techniques for electroblotted proteins	1986/7	A major breakthrough was the development of microsequencing techniques for electroblotted proteins	[41]
			[42]
Electrospray Ionization (ESI) and Matrix-assisted laser desorption/ionization (MALDI)	1989	ESI and MALDI first used to vaporize and ionize large molecules. This enabled transformation of proteins into the gas phase for MS analysis	[43]
			[44]
Surface-enhanced laser desorption/ionization (SELDI)	1993	First report of the use of SELDI for analysis of marcomolecules	[45]
Proteome and proteomics	1995	First use of the terms “proteome” and “proteomics” to denote the full complement of an organism’s proteins and their study, respectively	[18]
			[46]
			[47]
Isotope coded affinity tags (ICAT)	1999	First report of use of ICAT in a proteomic study	[48]
Reverse phase protein array	2001	First described in 2001	[49]
Stable isotopic labeling by amino acids in cell culture (SILAC)	2002/3	First reports of use of SILAC in proteomic studies	[50]
			[51]
Pharmacoproteomics	2002	First use of the term “pharmacoproteomics” in peer-reviewed literature to indicate the use of proteomics in the study of drug effects	[19]
			[20]
Isobaric tags for relative and absolute quantification (iTRAQ)	2004	First report of the use of iTRAQ in the quantification of Saccharomyces cerevisiae	[52]

**Table 2:** Early-phase pharmacoproteomic studies published in PubMed. Results of search conducted 18 November 2016.

PubMed Publication	Drug	Phase	Condition	Proteomic Objectives	Proteomic Analytics	Findings
Coleman et al. 2016 [54]	PepCan	1	HPV	Safety and efficacy of HPV vaccination, Proteomic analysis was used as exploratory endpoint to determine feasibility of biomarker identification	LC-MS/MS, LTQ-FT-Orbitrap	Differences in protein expression between baseline and post vaccination were detected. Feasibility of using the PBMC samples for proteomic analysis was established. Requirement to be consistent with the sample processing after blood draws was realized
Cheraghchi-Bashi et al. 2015 [55]	GSK2141795	1	Ovarian Cancer	Validating ovarian cancer proteomic signatures identified in preclinical xenograft and cell line studies	ELISA, Protein arrays	Proteomic signature was established as a predictive biomarker and could be used in patient stratification in larger studies. Importance of noninvasive methods to obtain samples for biomarker assessment was emphasized
Corcoran et al. 2015 [56]	Dabrafenib + Tramatenib	1	mCRC	Proteomic biomarker assessment for treatment of mCRC	Protein arrays	No correlation established between protein markers and mCRC treatment effects
Buscail et al. 2015 [57]	CYL-02	1	Pancreatic cancer	Safety, PK, and efficacy in pancreatic cancer. High throughput proteomic study conducted as exploratory endpoint	LC-MS/MS	Proteomic signature identified as predictive biomarker and correlated with good and poor treatment responders
Hare et al. 2015 [58]	Telaprevir in combination with peg-interferon and ribavirin	2	HCV	High throughput proteomic analysis conducted on samples from 3 phase-2 treatment studies for HCV	LC-MS/MS	Proteomic signature established as potential predictive biomarker. Proteomic analysis enhances understanding of biological mechanisms leading to response
Lee et al. 2014 [59]	Olaparib and carboplatin	1/2	Breast/ovarian cancer	Exploratory proteomic analysis for breast/ovarian cancer treatment efficacy	Protein arrays	pS209-eIF4E and FOXO3a may be predictive of response. Prospective studies are required for validation
Cardin et al. 2014 [60]	Erlotinib with sorafenib	2	Pancreatic adenocarcinoma	VeriStrat^®^ testing of pre-treatment samples to predict outcomes in treatment of pancreatic adenocarcinoma	MALSI-MS (VeriStrat)	Proteomic classification demonstrated correlation with clinical outcomes and could be useful in designing future therapeutic pancreatic cancer studies
Maitland et al. 2014 [61]	Cetuximab and Pemetrexed	2	NSCLC	Development of proteomic biomarkers for EGFR inhibitor efficacy in NSCLC	MALSI-MS (VeriStrat)	Serum proteomic markers may be predictive of NSCLC outcomes
Templeton et al. 2013 [62]	Everolimus	2	mCRPC	Proteomic analysis used to explore serum biomarkers in treatment of mCRPC	Immuno-histo-chemistry; hybrid LTQ-FT-MS	Proteomic biomarkers could be predictive of treatment outcomes but need further validation
Azad et al. 2013 [63]	Sorafenib and Bevacizumab	1	Solid tumors	Identifying proteomic biomarkers of response to treatment of solid tumors	Protein array; immune-histo-chemistry	Proteomic biomarkers that are potentially predictive of treatment effects were identified and will be used for stratification in larger studies
Stinchcombe et al. 2013 [64]	Gemcitabine and Erlotinib	2	NSCLC	Exploratory examination of the potential of proteomic test VeriStrat^®^ to predict treatment outcomes of NSCLC	MALSI-MS (VeriStrat)	VeriStrat^®^ can predict treatment outcomes in NSCLC patients
Akerley et al. 2013 [65]	Bevacizumab and Erlotinib	2	NSCLC	Prospective evaluation of proteomic serum biomarkers in the prediction of response to NSCLC treatment	MALSI-MS (VeriStrat)	Proteomic biomarkers (VeriStrat^®^) can be used for patient selection and are predictive of NSCLC treatment outcomes
Chinnaiyan et al. 2011 [66]	Vorinostat & Bevacizumab and CPT-11	1	Glioblastoma	Use of proteomic profiling to identify serum biomarkers of glioblastoma treatment outcomes. Serum proteomic profiling was an exploratory endpoint	Protein array	Proteomic analysis provided preliminary information on predictive and prognostic biomarkers (PFS and recurrence)
Jensen et al. 2011 [67]	Cetuximab and IMRT plus C12 heavy ion boost	2	ACC	Predict treatment efficacy in ACC. Well known markers for angiogenesis and tumorigenesis will be assessed from collected samples	ELISA	Not Applicable—description of an ongoing study protocol
Dalenc et al. 2010 [68]	Tipifarnib	2	Metastatic breast cancer	Identifying markers of therapeutic response in breast cancer patients treated with FTIs	SELDI-TOF, LTQ-FT-Orbitrap	Proteomic analysis identified a peptide of fibrinogen α that correlated with disease progression
Tabernero et al. 2010 [69]	Cetuximab	1	mCRC	Identifying biomarkers of cetuximab-responsive disease in plasma and tissue samples	Immuno-assays for 97 proteins	Candidate predictive biomarkers of response to cetuximab treatment were identified including inhibition of signaling proteins
Debucquoy et al. 2009 [70]	Cetuximab with chemo-radiotherapy	2	Rectal cancer	Identifying biomarkers predictive of response in plasma and tissue samples	Immune-assay	Proteomic profile of patients is predictive of disease-free survival in cetuximab-treated rectal cancer patients
Schilder et al. 2009 [71]	Cetuximab	2	Ovarian or peritoneal carcinoma	Prediction of response using proteomic serum biomarkers	ELISA and bead-based immune-assays	Serologic biomarkers were identified and patients with elevated levels are more likely to have earlier disease progression versus stable disease or partial remission
O'Byrne et al. 2007 [72]	Gefitinib and Rofecoxib	2	NSCLC	Identifying proteomic markers of response to EGFR TKIs	MALDI	Proteomic biomarkers were identified which could identify patients most likely to benefit from treatment from those with stable illness or progressive disease
Posadas et al. 2007 [73]	Imatinib	2	Ovarian cancer	Identifying proteomic biomarkers of response to treatment in tumor biopsies	Protein array	Though the study did not meet the primary endpoints of response to imatinib treatment, biomarker correlation with treatment was consistent with in vitro molecular signaling findings
Dragovich et al. 2006 [74]	Erlotinib	2	Gastric adenocarcinoma	Identifying plasma proteomic markers of response to erlotinib treatment	ELISA, SELDI	No biomarker correlations with treatment response were identified

HPV: Human Papilloma Virus; LC-MS/MS: Liquid chromatography tandem mass spectrometry; LTQ-FT-Orbitrap: Linear ion trap Fourier transform Orbitrap; mCRC: Metastatic colorectal cancer; PK: Pharmacokinetics; HCV: Hepatitis C virus; NSCLC: Non-small cell lung cancer; EGFR: Epidermal growth factor receptor; mCRPC: Metastatic castration-resistant prostate cancer; PFS: Progression-free survival; ACC: adenoid cystic carcinoma; IMRT: Intensity-modulated radiation therapy; SELDI-TOF: Surface-enhanced laser desorption/ionization time of flight; FTI: Farnesyltransferase inhibitor; TKIs: Tyrosine kinase inhibitors; MALDI: Matrix assisted laser desorption/ionization.

**Table 3:** Challenges of pharmacoproteomics applications in early-phase drug development.

Challenges in the Application of Pharmacoproteomics Approaches in Early-Phase Development	
**Methodological** [13]	Protocol and study design are complex Statistical analysis plan require specific expertise Incorporating into the protocol logistics for sample collection, handling, storage and processing Incorporating proteomic procedures into the consent process and protocol is complex due to additional information on sampling/biopsies, utilization of the analyses, and bio-banking Incorporation of surrogate and exploratory endpoints, and interim proteomic analyses
**Operational** [80,81,82]	Clinical sites require proteomic-related expertise to implement the protocols Clinical sites need infrastructure to support sample collection, processing, storage, and shipping Subject and site burden is increased in terms of time invested and labor requirements Robust analytical tools are required Enrollment can be challenging with additional sample requirements and privacy concerns In multicounty trials, sites may have different cultural values and regulations regarding bio-banking, exporting, and utilization of proteomic samples and data
**Regulatory** [82]	Regulatory burden increases as parallel regulatory strategy needs to be formed and there may be a need to comply with multiple sets of rules and regulations for biomarker processing Conducting global multicenter studies is complicated as countries may have different regulations on sample export to central laboratories Integrating different IRBs requirements on bio-banking and consenting
**Financial and legal** [82]	All the described challenges translate into substantial increase in financial burden Partners with relevant expertise on assay development and analytics have to be sought and relevant intellectual property agreements have to be established
**Ethical challenges** [83]	Privacy concerns regarding bio-banking and generated proteomic information Concerns regarding the interpretation of exploratory endpoints Ensuring informed consent process adequately covers proteomic analyses Handling subjects who refuse proteomic analyses or withdraw previously given consent

## Supplementary Materials

Supplementary materials can be found at www.mdpi.com/1422-0067/18/2/448/s1.
