# Supplementary material for: Integrating Pharmacoproteomics into Early-Phase Clinical Development: State-of-the-Art, Challenges, and Recommendations
Source: Int J Mol Sci. 2017 Feb 19;18(2):448. doi: 10.3390/ijms18020448 (PMC5343982; doi:10.3390/ijms18020448)
Supplement: Supplementary file 1 [file ijms-18-00448-s001.pdf]

Supplementary Materials: Integrating Pharmacoproteomics into Early-Phase Clinical Development: State-of-the-Art, Challenges, and Recommendations

Savita Nandal and Tal Burt

Table S1. Result of Clinical trials.gov.database search.

Number	NCT No.	Phase	Objective of Proteomic Biomarkers	Sponsor	Year	Therapeutic Area	Title
1	NCT02008994	2	Prognosis	Academia	2013	Oncology	Phase II study using genomic & proteomic profiling to influence treatments for patients with metastatic breast cancer
2	NCT00669773	2	Efficacy	Industry	2007	Skeletal system	This study will evaluate the ability of comparative proteomics to identify early biomarkers of muscle anabolism
3	NCT00669773	2	Prognosis biomarker validation	Academia	2008	Oncology	Validate gene expression and proteomic signatures predictive of treatment for response for breast cancer patient
4	NCT02783924	2	Pathophysiology	Academia	2014	Endocrinology	The GLOBAL vitamin d study. a genomic, transcriptomic, proteomic and metabolomic approach
5	NCT01728181	1/2	Efficacy safety	Academia	2013	Oncology	A Phase I/II study of tivozanib and erlotinib as initial treatment for metastatic non-small cell lung cancer assigned by VeriStrat® serum proteomic evaluation
6	NCT01383590	1	Biomarker development	Industry	2011	Immunology	Identification of potential biomarkers of peptide immunotherapy. part 1—proteomics analysis
7	NCT02470819	2	Efficacy	Academia	2014	Oncology	Genomic and proteomic profiling targets influenced treatment in metastatic breast cancer
8	NCT00550537	2	Prognosis	Academia	2007	Oncology	Proteomic profiling in predicting response in patients receiving erlotinib for stage IIIB, stage IV, or recurrent non-small cell lung cancer
9	NCT00198237	2	Efficacy	Collaboration	2003	Oncology	Genomic and proteomic analysis of docetaxel & capecitabine as primary chemo for stage ii-iii breast cancer
10	NCT01484080	1/2	Efficacy	Academia	2011	Oncology	Neoadjuvant paclitaxel versus BIBF 1120 priming followed by BIBF 1120 plus paclitaxel in early HER-2

							Negative breast cancer with proteomic and dynamic imaging correlates
11	NCT01962454	1	Efficacy	Industry	2013	Endocrinology	A study to evaluate a skeletal-muscle microbiopsy technique with dynamic proteomic measurement in healthy male volunteers
12	NCT00212069	2	Exploratory	Academia	2004	Oncology	Serum proteomics to predict gemcitabine sensitivity in breast cancer
13	NCT01097902	1	Predicts response or efficacy	Academia	2010	Pain	Study of experimental models of pain and inflammation
14	NCT02040441	2/3	Diagnostic, mechanism, efficacy and quantification	Academia	2013	endocrinology	Proteomic prediction and renin angiotensin aldosterone system inhibition prevention of early diabetic nephropathy in TYpe 2 diabetic patients with normoalbuminuria (PRIORITY)
15	NCT00212095	2	Safety	Academia	2005	Oncology	Docetaxel combined with ketoconazole in treatment of breast cancer
16	NCT00714246	1/2	Efficacy or treatment response	Collaboration	2008	Oncology	Bortezomib (PS-341) in combination with carboplatin and docetaxel for patients with advanced non-small cell lung cancer
17	NCT00953472	1	Efficacy	Academia	2009	Cardiovascular system	B-type natriuretic peptide (bnp) in human hypertension
18	NCT00114738	2	Efficacy	Academia	2005	Oncology	EPOCH-R chemotherapy plus bortezomib to treat mantle cell lymphoma
19	NCT00817531	2	Efficacy	Academia	2008	Oncology	Efficacy study of dasatinib in locally advanced triple-negative breast cancer patients
20	NCT00039585	2	Efficacy and toxicity	Academia	2002	Oncology	Imatinib mesylate in treating patients with refractory or relapsed ovarian epithelial, fallopian tube, or primary peritoneal cancer, or ovarian low malignant potential tumor
21	NCT00884767	2	Safety	Academia	2007	Oncology	Biomarkers in predicting neurotoxicity in patients with colorectal cancer receiving Oxaliplatin
22	NCT00867334	1/2	Safety and efficacy	Collaboration	2009	Oncology	New individualized therapy trial for metastatic colorectal cancer (NITMEC)
23	NCT02836847	2	Predictive	Academia	2016	Oncology	Molecularly target therapy with gemox in advanced or recurrent extrahepatic cholangiocarcinoma and gallbladder carcinoma
24	NCT00095459	1	Efficacy	Academia	2004	Oncology	BAY 43-9006 (sorafenib) and bevacizumab (avastin) to treat solid tumors

25	NCT00212082	2	Efficacy and safety	Academia	2002	Oncology	Gene expression profiles in predicting chemotherapy response in breast cancer
26	NCT00049556	2	Efficacy and safety	Academia	2002	Oncology	Gefitinib in treating patients with cervical cancer
27	NCT01548924	1	Efficacy and safety	Academia	2012	Oncology	Determination of dose of antiangiogenic multitargeted DOVITINIB (TKI258) plus paclitaxel in patients with solid tumors
28	NCT00445549	2	Efficacy	Academia	2007	Oncology	Vandetanib to treat women with ovarian, fallopian tube, or primary peritoneal cancer
29	NCT00950820	2	Efficacy and safety	Academia	2009	Oncology	Study to evaluate the effects of panitumumab if combined with chemotherapy for 2nd treatment of colorectal cancer (VOXEL)
30	NCT01012609	2	Efficacy	Academia	2009	Oncology	External beam radiation therapy and cetuximab followed by irinotecan and cetuximab for children and young adults with newly diagnosed diffuse pontine tumors and high-grade astrocytomas
31	NCT02891980	1	Efficacy	Academia	2016	Immunology/healthy volunteers	MVA-BN®-Filo and Ad26.ZEBOV vaccines in healthy volunteers
32	NCT00619541	2	Efficacy and safety	Academia	2007	Oncology	Study of sorafenib and infusional 5-fluorouracil in advanced hepatocellular carcinoma (HCC)
33	NCT00619996	2	Efficacy and safety	Academia	2007	Oncology	Study of sorafenib and docetaxel in metastatic prostate cancer
34	NCT00251225	2	Efficacy	Collaboration	2005	Oncology	A study of imatinib and docetaxel in prostate cancer
35	NCT00937417	1	Efficacy	Academia	2008	Oncology	S0716 vandetanib and docetaxel in treating patients with advanced solid tumors
36	NCT00354978	2	Efficacy	Collaboration	2004	Oncology	Study of FOLFIRI Plus bevacizumab in colorectal cancer patients
37	NCT00807950	2	Refine selection of patients	Academia	2008	Oncology	Phase II study of simvastatin in primary breast cancer; test of its potential selectivity on basal subtype breast cancer
38	NCT00866762	2	Mechanistic	Academia	2009	Oncology	A study of the efficacy of MK-0683 in patients with polycythaemia vera and essential thrombocythaemia
39	NCT02557529	2	Exploratory	Academia	2015	Oncology	Progressive resistance training in head and neck cancer patients during concomitant chemoradiotherapy
40	NCT00877500	2	Efficacy	Collaboration	2009	Oncology	Randomized study of ixabepilone versus observation in patients with significant residual disease
41	NCT00111397	1	Exploratory	Academia	2005	Infection	Adjuvant cytokine therapy to treat pulmonary mycobacterium avium complex infection

42	NCT02406794	1/2	Safety and efficacy	Academia	2016	Oncology	Effect of kinesiotaping for the treatment of hormone-induced myalgia in women given breast cancer survivors (KITMYCAN)
43	NCT02582827	1	Exploratory	Industry		Oncology	QUILT-3.014: a trial of ABI-011 administered weekly in patients with advanced solid tumors or lymphomas
44	NCT01290172	1	Exploratory	Academia	2010	Transplantation	Evaluation of efficacy and safety of somatostatin used as inflow modulator in liver transplantation
45	NCT01215578	2	Predictive	Academia	2008	Oncology	Predictive biomarkers of response to sunitinib in the treatment of poorly-differentiated neuro-endocrine tumors (NET)
46	NCT00811031	2	Efficacy	Industry	2008	Oncology	Preoperative docetaxel for localized progressive castration-resistant prostate cancer (CRPC)
47	NCT02921997	2	Efficacy	Academia	2016	Immunology	Immunogenicity of monovalent inactivated influenza A/H7N9 virus vaccine
48	NCT00672009	2	Efficacy	Collaboration	2008	Oncology	A study of ixabepilone before surgery for high-risk localized prostate cancer
49	NCT00255463	2	Exploratory	Industry	2004	Oncology	Phase II neoadjuvant ER+/PgR + Arimidex ± Iressa study
50	NCT00382694	2	Prognostic	Academia	2005	Oncology	Fludarabine added to induction treatment in untreated multiple myeloma patients
51	NCT00121953	2 / 3	Exploratory	Academia	2005	Gynaecology	Effect of rosiglitazone on peritoneal cytokines in women with endometriosis
52	NCT01233492	1	Exploratory	Academia	2007	Oncology	Boron phenylalanine with or without mannitol in treating patients with glioblastoma multiforme
53	NCT00466505	2	Safety/efficacy	Academia	2005	Oncology	Cetuximab and celecoxib for metastatic colorectal cancer or colorectal cancer that cannot be removed by surgery
54	NCT01047293	1/2	Efficacy and safety	Collaboration	2010	Oncology	RAD001, FOLFOX and bevacizumab in treatment of colorectal carcinoma
55	NCT01023477	1/2	Efficacy	Academia	2009	Oncology	Study of the efficacy of chloroquine in the treatment of ductal carcinoma in Situ (The PINC Trial)
56	NCT02520115	1	Diagnostic	Academia	2015	Oncology	Folate receptor in diagnosing ovarian cancer using serum samples from patients with newly diagnosed pelvic mass or previously diagnosed ovarian cancer
57	NCT00115661	1	Exploratory	Academia	2005	Gynaecology	Use of rosiglitazone in the treatment of endometriosis
58	NCT00090545	2	Biological effect	Academia	2004	Oncology	Phase II study of BAY 43-9006 (Sorafenib) in metastatic, androgen-independent prostate cancer
59	NCT00416130	1/2	Predictive marker	Collaboration	2007	Oncology	Phase I/II clinical trial of vorinostat in patients with recurrent and/or metastatic breast cancer

60	NCT00674557	2	Exploratory	Academia	2008	Oncology	Exemestane with or without atn-224 in treating postmenopausal women with recurrent or advanced breast cancer
61	NCT00601289	2	Exploratory	Academia	2009	Oncology	Temozolomide in treating patients with invasive pituitary tumors
62	NCT00972933	2	Efficacy	Collaboration	2009	Oncology	Immunogenicity and biomarker analysis of neoadjuvant ipilimumab for melanoma
63	NCT01282502	1	Efficacy	Academia	2011	Oncology	Midostaurin (PKC412) for locally advanced rectal cancer
64	NCT02434627	1	Exploratory	Academia	2015	Skeletal system	Sodium nitrate for muscular dystrophy
65	NCT00324597	1	Exploratory	Academia	2005	Oncology	AMG 706 and gemcitabine in treating patients with advanced solid tumors or lymphoma
66	NCT00445341	1/2	Exploratory	Academia	2007	Oncology	Flavopiridol to treat relapsed mantle cell lymphoma or diffuse large b-cell lymphoma
67	NCT00329914	2	Efficacy	Academia	2006	Gynaecology	Does progesterone prevent very preterm delivery in twin pregnancies
68	NCT01129453	1	Exploratory	Academia	2010	Infection	Safety and immunogenicity of CVD 1902 Oral attenuated vaccine to prevent *S. paratyphi* A infection
69	NCT02514941	1	Exploratory	Academia	2007	Obesity	Bioavailability of paracetamol, amoxicillin and talinolol before, immediately and one year after gastric bypass operation
70	NCT00645710	1/2	Exploratory	Academia	2005	Oncology	Hepatic arterial infusion of floxuridine, gemcitabine hydrochloride, and radiolabeled monoclonal antibody therapy in treating liver metastases in patients with metastatic colorectal cancer previously treated with surgery
71	NCT00770471	1/2	Exploratory	Academia	2009	Oncology	ABT-888, radiation therapy, and temozolomide in treating patients with newly diagnosed glioblastoma multiforme
72	NCT00098072	1	Exploratory	Academia	2004	Lung and respiratory	Endothelial cell dysfunction in pulmonary hypertension
73	NCT00536874	2	Efficacy	Academia	2007	Oncology	Gemcitabine and oxaliplatin in treating patients with pancreatic cancer that can be removed by surgery
74	NCT00354679	2	Efficacy	Academia	2006	Oncology	Irinotecan, cisplatin, bevacizumab, radiation therapy, and surgery in treating patients with locally advanced esophageal cancer
75	NCT01338753	2	Efficacy	Collaboration	2009	Oncology	Study to evaluate markers of response in locally advanced breast cancer (IMAGING)

76	NCT00433485	1	Exploratory	Academia	2008	Oncology	Topical sirolimus in patients with basal cell nevus syndrome and in healthy participants
77	NCT00851552	2	Exploratory	Academia	2009	Oncology	Bortezomib, doxorubicin hydrochloride liposome, and rituximab in treating patients with diffuse large b-cell lymphoma that has relapsed or not responded to treatment
78	NCT00602082	2	Exploratory	Academia	2005	Oncology	Capecitabine and streptozocin with or without cisplatin in treating patients with unresectable or metastatic neuroendocrine tumors
79	NCT00389922	1	Safety	Academia	2005	Oncology	Lapatinib and vinorelbine in treating patients with advanced solid tumors
80	NCT00391170	2	Exploratory	Academia	2006	Transplantation	Dexamethasone to prevent oral chronic graft-versus-host disease
81	NCT01263145	1	Exploratory	Academia	2011	Oncology	MK2206 and paclitaxel in treating patients with locally advanced or metastatic solid tumors or metastatic breast cancer
82	NCT00546897	2	Efficacy	Academia	2007	Oncology	Lenalidomide in older patients with acute myeloid leukemia without chromosome 5q abnormalities
83	NCT00108836	2	Exploratory	Industry	2005	CNS	Efficacy, safety and tolerability of XBD173 in patients with generalized anxiety disorder
84	NCT00971230	1/2	Exploratory	Academia	2009	Infection	A pilot study of pre-exposure prophylaxis (PrEP) to evaluate safety, acceptability, and adherence in at-risk populations in Kenya, Africa
85	NCT01504542	2	Exploratory	Industry	2011	Oncology	Immune response and safety of hs110 vaccine in combination with erlotinib in patients with non-small cell lung cancer
86	NCT00674414	2	Efficacy	Industry		Oncology	Trastuzumab with or without everolimus in treating women with breast cancer that can be removed by surgery
87	NCT00722969	2	Efficacy	Academia	2007	Oncology	Erlotinib and sorafenib in chemonaive patients with locally advanced or metastatic non small cell lung cancer
88	NCT00548899	2	Efficacy	Collaboration	2007	Oncology	Neoadjuvant chemotherapy including sorafenib in women with previously untreated primary breast cancer (SOFIA)
89	NCT00375310	1	Efficacy	Collaboration	2006	Oncology	Phase I study of gemcitabine, sorafenib and Radiotherapy in patients with unresectable pancreatic cancer

90	NCT02502006	1	Efficacy	Academia	2015	Pain	Variability in response to non-steroidal anti-inflammatory drugs
91	NCT01425580	2	Efficacy	Academia	2012	endocrinology	Liraglutide and heart failure in type 2 diabetes
92	NCT01110785	2	Efficacy	Academia	2010	Oncology	Simvastatin and panitumumab in treating patients with advanced or metastatic colorectal cancer
93	NCT00967031	2	Efficacy	Academia	2009	Oncology	Lapatinib ditosylate and capecitabine in treating patients with stage iv breast cancer and brain metastases
94	NCT00331058	1	Efficacy	Industry	2006	Lung and respiratory	Comparison of molecular targets in mild to severe asthmatics and healthy subjects
95	NCT01328171	2	Efficacy	Collaboration	2011	Oncology	FOLFOXIRI with or without panitumumab in metastatic colorectal cancer (VOLFI) (VOLFI)
96	NCT01251536	2	Efficacy	Collaboration	2010	Oncology	Cetuximab standard or dose escalation in first line colorectal cancer (everest2)
97	NCT00794274	2	Efficacy	Academia	2008	Lung and respiratory	The efficacy and safety of CC-10004 in chronic cutaneous sarcoidosis
98	NCT02772679	1	Efficacy	Academia	2016	Endocrinology	T1DM immunotherapy using polyclonal tregs + IL-2 (TILT)
99	NCT00540007	2	Exploratory	Industry	2007	Oncology	Lenalidomide in relapsed or refractory classical hodgkin lymphoma
100	NCT00931346	1/2	Efficacy	Academia	2009	Infection	A pilot study of pre-exposure prophylaxis (PrEP) to evaluate safety, acceptability, and adherence in at-risk populations in Uganda, Africa
101	NCT00820547	2	Efficacy	Industry	2009	Oncology	Efficacy and tolerance study of bevacizumab in her2-inflammatory breast cancer patients (beverly1)
102	NCT00471588	1	Exploratory	Industry	2006	Central Nervous System	Characterize the modulatory effects of dopamine D2/D3 receptor agonist and antagonist drugs on compulsive behaviors
103	NCT00073073	2	Exploratory	Academia	2003	Oncology	Exemestane and celecoxib in postmenopausal women at high risk for breast cancer
104	NCT02598557	2	Exploratory	Academia	2016	Oncology	Alternative dosing of exemestane before surgery in treating postmenopausal patients with stage 0-ii estrogen positive breast cancer
105	NCT01711398	1/2	Efficacy	Industry	2012	Oncology	Dose-finding adaptive phase I/IIa study to assess safety, tolerability, pharmacokinetics and preliminary efficacy of IPP-204106N on advanced solid tumors (IP N02)

106	NCT01245985	2	Exploratory	Academia	2010	Oncology	TPF followed by cetuximab and imrt plus carbon ion boost for locally advanced head and neck tumors (TPF-C-HIT)
107	NCT01714037	1	Exploratory	Industry	2012	Oncology	A clinical study on the safety and efficacy of debio 0932 in combination with standard of care in patients with non-small cell lung cancer [NSCLC] (HALO)
108	NCT01240460	1	Efficacy	Industry	2011	Oncology	Exploratory study of XL765 (SAR245409) or XL147 (SAR245408) in subjects with recurrent glioblastoma who are candidates for surgical resection
109	NCT00105950	2	Efficacy	Industry	2005	Oncology	Study of lapatinib in patients with relapsed or refractory inflammatory breast cancer
